# Effect of Consuming Oat Bran Mixed in Water before a Meal on Glycemic Responses in Healthy Humans—A Pilot Study

**DOI:** 10.3390/nu8090524

**Published:** 2016-08-26

**Authors:** Robert E. Steinert, Daniel Raederstorff, Thomas M. S. Wolever

**Affiliations:** 1 DSM Nutritional Products Ltd., R & D Human Nutrition and Health, Basel 4057, Switzerland; daniel.raederstorff@dsm.com; 2 Glycemic Index Laboratories, Inc., Toronto, ON M5C 2N8, Canada; thomas.wolever@utoronto.ca

**Keywords:** postprandial glycemia, dietary fibre, blood glucose, type 2 diabetes mellitus, preload

## Abstract

Background: Viscous dietary fibers including oat β-glucan are one of the most effective classes of functional food ingredients for reducing postprandial blood glucose. The mechanism of action is thought to be via an increase in viscosity of the stomach contents that delays gastric emptying and reduces mixing of food with digestive enzymes, which, in turn, retards glucose absorption. Previous studies suggest that taking viscous fibers separate from a meal may not be effective in reducing postprandial glycemia. Methods: We aimed to re-assess the effect of consuming a preload of a commercially available oat-bran (4.5, 13.6 or 27.3 g) containing 22% of high molecular weight oat β-glucan (O22 (OatWell^®^22)) mixed in water before a test-meal of white bread on glycemic responses in 10 healthy humans. Results: We found a significant effect of dose on blood glucose area under the curve (AUC) (*p* = 0.006) with AUC after 27.3 g of O22 being significantly lower than white bread only. Linear regression analysis showed that each gram of oat β-glucan reduced glucose AUC by 4.35% ± 1.20% (*r* = 0.507, *p* = 0.0008, *n* = 40) and peak rise by 6.57% ± 1.49% (*r* = 0.582, *p* < 0.0001). Conclusion: These data suggest the use of oat bran as nutritional preload strategy in the management of postprandial glycemia.

## 1. Introduction

In current obesogenic societies with many people having mild or moderate hyperglycemia, postprandial blood glucose patterns account for the majority of variability of overall glycemic control [1,2]. This is not surprising considering that most individuals spend perhaps only about three to four hours before breakfast in a truly fasted state [1,3]. Dietary means to lower postprandial glycemic responses are, thus, urgently needed for the prevention of Type 2 Diabetes mellitus (T2DM). Viscous dietary fibers including high molecular weight (HMW) oat β-glucan are one of the most effective classes of functional food ingredients for reducing postprandial glucose [4]. The mechanism of action is thought to be their ability to increase the viscosity of the contents of the upper gastrointestinal (GI) tract and, hence, slow gastric emptying (GE) [5]. The rate of GE has a substantial impact on postprandial glycemia by determining glucose absorption and incretin hormone secretion [6,7]. In addition, an increase in viscosity of GI contents reduces the rate of digestion of starch by pancreatic amylase and the rate of absorption of glucose in the small intestine by increasing the thickness of the unstirred water layer [5].

Previous studies suggest that taking viscous fibers separate from a main meal may not be effective in reducing postprandial glycemia [8,9]. One potential solution to this problem may be to consume, before eating, a fiber “preload”, which develops viscosity slowly, so that it can be consumed in a palatable form, and remains liquid in the stomach long enough to be able to mix effectively with the main meal, but becomes viscous by the time the stomach starts to empty the meal. OatWell^®^22 (O22) is a commercially available oat-bran including 44% dietary fiber and 22% HMW oat β-glucan, which forms a palatable drink when mixed with water and which becomes viscous after several minutes. Therefore, we evaluated the dose-response effect of O22 mixed in water and consumed before a white bread meal on glycemic responses in healthy humans.

## 2. Subjects and Methods

### 2.1. Subjects

Ten healthy normal-weight, overweight and obese subjects (5 male/5 female, mean age (years): 48.0 ± 15.3 (range 22–65), BMI (kg/m^2^): 29.5 ± 4.4 (range 23.2–36.9)) were studied using an open-label, randomized block design. The study was performed according to accepted standards and the Declaration of Helsinki. Ethical approval was obtained from the Western Institutional Review Board, and the study was registered as a clinical trial with clinical trials.gov (registration number NCT02801916). Written informed consent was obtained from all participants.

### 2.2. Study Outline

Each subject underwent 4 treatments on separate days, with each subject performing up to 3 tests per week separated by at least one day. On each test day, subjects came to the laboratory in the morning after a 10–14 h overnight fast. Two fasting blood samples (*t* = −5 min and *t* = 0 min) were obtained by finger-prick. After the first fasting blood sample, subjects consumed a preload consisting of 200 mL water either alone or mixed with 4.5, 13.6 or 27.3 g of O22 containing 0.9, 2.6, and 5.3 g of oat β-glucan, respectively (DSM Nutritional Products, Table 1). After the second fasting blood sample (*t* = 0 min), subjects were asked to consume a test meal consisting of a portion (119 g) of white bread containing 50 g available carbohydrate (Table 1). The time taken to consume the bread was between 7 and 12 min. Further blood samples were obtained at 15, 30, 45, 60, 90 and 120 min after meal onset. Subjects remained seated quietly during the 2 h of the test. After the last blood sample was obtained, subjects were offered a snack and then permitted to leave.

### 2.3. Measurements

After consuming the test meal, subjects rated the palatability of the test meal using a visual analogue scale consisting of a 100-mm line anchored at the left end by “very unpalatable” and at the right end by “very palatable”. Subjects made a vertical mark along the line to indicate their perceived palatability. The distance from the left end of the line to the mark made by the subject is the palatability rating; the higher the value, the higher the perceived palatability.

Blood samples (2–3 drops each) were collected into 5 mL tubes containing ~500 μg sodium fluoride and 400 μg potassium oxalate. The samples were mixed and refrigerated immediately during the testing session. After completion of the test session, samples were stored at −20 °C prior to glucose analysis. Blood glucose analysis, using a YSI (Yellow Spring Instruments, Yellow Springs, OH, USA) analyzer, took place within five days of collection.

### 2.4. Data and Statistical Analysis

The primary endpoint was incremental area under the blood glucose curve (AUC) which was calculated using the trapezoid rule, ignoring area beneath the baseline. Baseline blood glucose was calculated as mean of values obtained at *t* = −5 min and *t* = 0 min. AUC and peak rise were analyzed using repeated-measures analysis of variance (RMANOVA) examining for the main effects of treatment dose. In case of significant heterogeneity, differences among different doses were tested using Tukey’s test to adjust for multiple comparisons. AUC and peak rises for each dose of O22 were expressed relative to the AUC or peak rise after the test of white bread alone taken by the same subject. The glycemic response of 1 subject with a relative response after the 13.6 g dose of 2.55 × SD above the mean was considered to be an outlier and the data removed; the values were replaced using a procedure described by Snedecor and Cochran [10], and the error degrees of freedom in RMANOVA reduced by one. Blood glucose concentrations at each time were subjected to RMANOVA examining for the main effects of time and treatment and the time × treatment interaction; after demonstrating a highly significant time × treatment interaction (*p* = 1.2 × 10^−17^), blood glucose concentrations at each time were subjected to RMANOVA followed by Tukey’s test, as described above for AUC. Differences were considered to be statistically significant if 2-tailed *p* < 0.05.

## 3. Results

**Figure 1:**
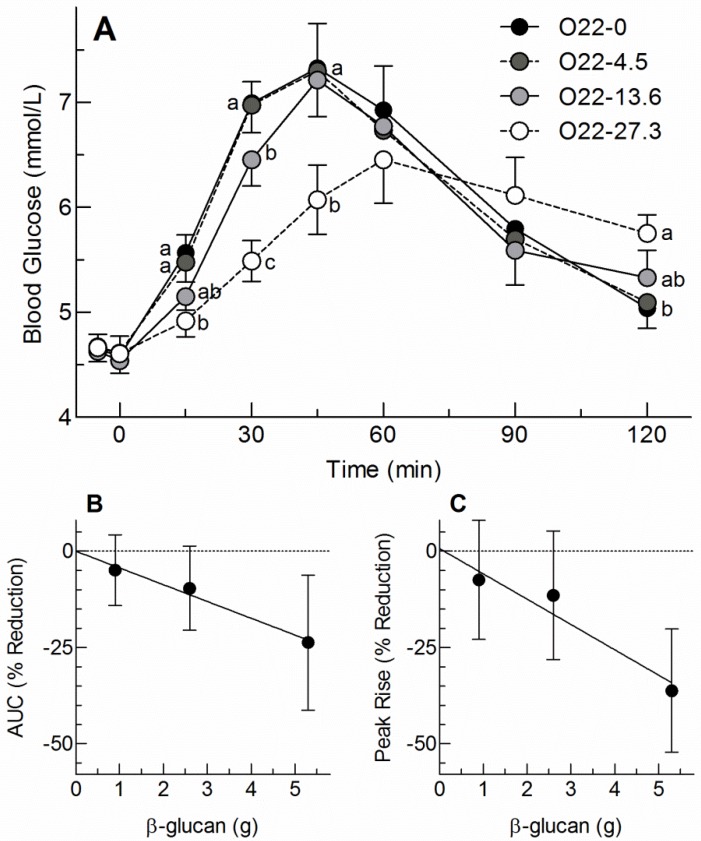
Panel (**A**): Blood glucose concentrations after taking 0, 4.5, 13.6 and 27.3 g, respectively, of OatWell^®^22 (O22-0, O22-4.5, O22-13.6 and O22-27.3) at −5 min followed by 50 g available carbohydrate from white bread at 0 min. Values are means ± SEM for *n* = 10 subjects. ^a–c^ Means at the same time containing different letters within the superscripts differ significantly by Tukey’s test *p* < 0.05; (**B**,**C**): Percentage reduction from control in incremental areas under the curve (AUC); (**B**) and peak rise in blood glucose; (**C**) after taking 0, 4.5, 13.6 and 27.3 g of OatWell^®^22 (containing 0, 0.9, 2.6 and 5.3 g oat β-glucan, respectively) at −5 min followed by 50 g available carbohydrate from white bread at 0 min. Values are means ± 95% confidence interval for *n* = 9 or 10 subjects (after excluding outlying values).

There were significant differences in blood glucose concentration among doses at 15 (*p* = 0.0015), 30 (*p* < 0.0001), 45 (*p* = 0.0003) and 120 min (*p* = 0.0004). At 15 min, blood glucose concentration after 27.3 g was significantly lower than those after 0 g and 4.5 g. At 30 min, blood glucose after the 27.3 g dose was significantly lower than after 13.6 g, which, in turn, was significantly lower than the 0 and 4.5 g doses. At 45 min blood glucose concentration after 27.3 g of O22 was significantly lower than those after the 0, 4.5 and 13.6 g doses. At 120 min, blood glucose after 27.3 g of O22 was significantly higher than that after the 0 and 4.5 g doses (Figure 1A). There was a significant effect of dose on blood glucose AUC (*p* = 0.006) with AUC after 27.3 g of O22 (141 ± 21 mmol × min/L) being significantly lower than both the 4.5 g and 0 g doses (174 ± 1 7 and 185 ± 18 mmol × min/L, respectively) and the AUC after 13.6 g being intermediate (167 ± 19 mmol × min/L). When AUC was expressed relative to that of bread alone, the relative responses for the 4.5, 13.6 and 27.3 g doses of O22, respectively, were 95.0% ± 4.1%, 90.3% ± 4.7% and 76.3% ± 7.7% with the only significant reduction being seen with the highest dose. However, linear regression analysis showed that each gram of oat β-glucan reduced glucose AUC by 4.35% ± 1.20% (*r* = 0.507, *p* = 0.0008, *n* = 40; Figure 1B). There was also a significant effect of dose for blood glucose peak rise (*p* < 0.0001) with the peak rise after 27.3 g of O22, 1.96 ± 0.29 mmol/L, being significantly lower than that after all the other doses (3.07 ± 0.25, 2.83 ± 0.30 and 2.72 ± 0.30 g for the 0, 4.5 and 13.6 g doses of O22, respectively). When peak rise was expressed relative to that for bread alone, the relative responses for the 4.5, 13.6 and 27.3 g doses of O22, respectively, were 92.5% ± 6.8%, 88.5% ± 7.2% and 63.8% ± 7.1% with the only significant reduction being seen with the highest dose. However, linear regression analysis showed that each gram of oat β-glucan reduced glucose peak rise by 6.57% ± 1.49% (*r* = 0.582, *p* < 0.0001, *n* = 40; Figure 1C).

There was a significant main effect of dose on palatability, with all 3 doses of O22 rated as being less palatable than white bread alone (palatability in mm: 71 ± 7 for white bread alone; 38 ± 9 for 4.5 g O22; 33 ± 10 for 13.6 g O22; 31 ± 9 for 27.3 g O22; *p* = 0.0003). Although palatability tended to decrease as the dose increased, this difference was not significant. There was no significant relationship between palatability and glucose AUC (expressed as % of that after white bread) for 4.5 g (*r* = 0.439, *p* = 0.20, *n* = 10), 13.6 g (*r* = 0.502, *p* = 0.14, *n* = 10) or 27.3 g (*r* = 0.398, *p* = 0.25, *n* = 10) doses of O22.

No adverse effects were observed.

## 4. Discussion

We demonstrated that consuming a commercially available oat-bran containing 22% of HMW oat β-glucan mixed in water before a white bread meal significantly lowers postprandial glycemia in a dose dependent manner with 27.3 g of O22 being significantly lower than white bread only. Linear regression analysis, in addition, showed that each gram of oat β-glucan reduced glucose AUC by ~4% and peak rise by ~7%. Given that the magnitude of this reduction would be similar in patients with T2DM, these data may have considerable implications for nutritional strategies in the management of diabetes, however, this concept warrants further investigation. The mechanism of action of oat beta-glucan to reduce postprandial glycemia is well established. In their native form, oat beta-glucan consists of very high molecular weight polysaccharides that exhibit high viscosities at low concentrations [11]. An increase in viscosity of a meal bolus in the stomach delays gastric emptying and reduces mixing of food with digestive enzymes. This retards absorption of glucose making oat β-glucan one of the most effective classes of functional food ingredients to reduce postprandial blood glucose and insulin responses [4,5].

Previous studies have suggested that taking viscous fibers, such as psyllium or guar gum, separate from a main meal may not be effective in reducing postprandial glycemia [12,13]. In contrast, the concept of a “preload” that refers to administration of a small load of nutrient at a fixed interval before a main meal to lower postprandial blood glucose via the slowing of GE has been confirmed several times [14,15,16]. While in the classical sense of the preload concept, the slowing of GE is related primarily to a nutrient-induced neuroendocrine feedback [14,15,16], the present findings suggest that the preload concept also applies to fibers such as oat beta-glucan that delay GE via increases in meal bolus viscosity. It requires consideration, however, that the oat bran also contained about 20% protein that may have contributed to the slowing of GE via neuroendocrine feedbacks. In addition, the protein may have lowered postprandial blood glucose independently of the preload effect because a reduction in postprandial blood glucose was observed also in studies with protein ingested simultaneously with carbohydrate-rich meals [17]. The discrepancy between the current results and those of previous investigations mentioned above suggests that proper control of method of administration of the preload such as timing (i.e., interval between preload administration and meal onset) and the physicochemical properties of the fiber at meal onset, as well as other macronutritional components, are important. Ideally the fiber preload remains liquid in the stomach long enough to be able to mix effectively with the meal, but becomes viscous by the time the stomach starts to empty the meal.

The present results are in line with the health claim that has previously been evaluated by the European Food Safety Authority (EFSA) and authorized by the European commission for β-glucans from oats and barley and reduction of postprandial glycemia [18]. The conditions of use state that in order to obtain the claimed effect, 4 g of beta-glucans for each 30 g of available carbohydrates should be consumed per meal. Our results further indicate that lower doses of HMW oat β-glucan may be sufficient to impact postprandial glycemia when consumed in a suitable manner (e.g., several smaller doses/day, correct timing/preload paradigm). This approach is in line with a recent systematic review by Tosh et al. [4], including 119 treatments from 34 publications that finds that the efficacy of oat and barley β-glucan in lowering postprandial blood glucose is more strongly related to β-glucan content alone than to the ratio of β-glucan/available carbohydrate. In addition, when authors actually calculated a ratio, 4 g of β-glucan per meal were found to be sufficient to reduce post-prandial blood glucose in a clinical relevant amount for meals with up to 80 g of available carbohydrate.

A limitation of this pilot trial that requires consideration is that it was not powered to detect small differences seen with the lower doses used here (i.e., 4.5 and 13.6 g). We only found a significant effect with 27.3 g of O22, however, the results show that the expected reduction in AUC and peak rise for the 13.6 g dose were 11.3% and 17.1%, respectively. Therefore, based on the results of this study, 27 subjects would be required to have 80% power to detect an 11.3% reduction in AUC and 14 subjects to detect a 17.1% reduction in peak rise. The additional energy intake associated with an oat bran preload should also be considered if using such a strategy over the long term, althoughsubjects usually tend to compensateat least in part, for extra energy by eating less at a subsequent ad libitum meal. There is good evidence for particularly viscous fibers to reduce appetite and eating. For example, a recent systematic review including 58 original studies by Wanders et al. [19] reports a reduction in appetite perception by 7.4% over a 4 h time interval when dietary fiber is added as part of a preload. Similarly, a recent meta-analysis including 6 human studies shows that polydextrose fiber as part of a mid-morning preload reduces energy intake at a subsequent ad libitum meal at lunch time by 12.5% [20]. Finally, there is evidence from epidemiological studies that show associations between high fiber intakes and weight loss [21,22].

In conclusion, consuming O22 mixed in water before a meal reduces glycemic response in a dose-dependent manner with each gram of oat β-glucan reducing glucose AUC by about 4%. This suggests the use of O22 as a nutritional preload strategy in the management of postprandial glycemia.

## Figures and Tables

**Table 1:** Nutrient content of test meal ingredients.

Test Meal	Energy (kcal)	Weight (g)	Protein (g)	Fat (g)	tCHO ^1^ (g)	Fibre (g)		avCHO ^1^ (g)
						Total	β-Glucan	
White Bread ^1^	245	119	9.0	1.0	52.6	2.6	0	50.0
OatWell^®^22 ^2^	13.1	4.5	1.0	0.2	3.0	2.2	0.9	0.8
	39.7	13.6	3.1	0.6	8.8	6.5	2.6	2.3
	79.7	27.3	6.2	1.1	17.7	13.1	5.3	4.6

^1^ Baked at Glycemic Index Laboratories using an automatic bread maker; values represent the mean of 5 proximate analyses performed by Gelda Scientific, Mississauga, ON, Canada; ^2^ Nutritional analysis, including β-glucan content, was performed by SGS Institute Fresenius GmbH, Im Maisel, Taunusstein, Germany.
